# A Predictive Model of Apartment-Living Based on Socio-Economic and Demographic Factors With Health-Based Approach in Iran

**DOI:** 10.5539/gjhs.v7n3p324

**Published:** 2015-03-10

**Authors:** Pezhman Bagheri, Vajihe Armanmehr, Noorallah Moradi, Mahdi Moshki

**Affiliations:** 1 Department of Social Medicine, faculty of medicine, Fasa University of Medical Science, Fasa, Iran; 2 Social Development & Health Promotion Research Center, Gonabad University of Medical Science, Gonabad, Iran; 3 Department of Public Health, School of Health; Social Development & Health Promotion Research Center, Gonabad University of Medical Science, Gonabad, Iran

**Keywords:** apartment-living, demographic factors, health behavior, Iran, lifestyle, predictive model, socioeconomic factors

## Abstract

**Objective:**

Due to importance and progressive growth of apartment-living phenomenon in the world today, it is essential to survey the different dimensions of this modern lifestyle. The aim of this study is to predict rate of apartment-living based on the different predicted variables of socio-economic and demographic factors with approach to different health aspects.

**Methods:**

A descriptive- analytic study was carried out between 600 apartment and 800 non-apartment residents in the Shiraz (Southern Region of Iran) through multi-stage cluster sampling during the year 2011. The statistical analysis was performed on the obtained data using multi-variable logistic regression as well as ANOVA analysis.

**Result:**

The rate for apartment-living in above 30 years old age group was 8.31 times more than 15-30 years old, 9.6 times more in employed vs. unemployed; 6.57 and 9.49 times more in families with average and high monthly incomes, respectively, vs. family with low monthly income; 8.73 times more in owner sub-group vs. renter sub-group; and 1.30 times more in people living lonely than those living with family. People living in an apartment are in poor health status considering physical, mental and social aspects.

**Conclusion:**

Based on the results, it is very important that policy makers in urban areas consider the determinative role of socio-economic and demographic factors, which are involved in selecting apartment-living lifestyle by urban residents and also are effective on health.

## 1. Introduction

Urbanization is a highly complex phenomenon, yet affecting the lives of individuals, families, communities and countries, which can be named as the main determinant of health in the current century. Urbanization, and its progressive development, has a great impact on economy and environment of a region. Life style and attitude of the citizens are subject to large changes due to progressive urbanization. Unplanned and rapid expansion of urban areas in many countries lead to marginalization, slum areas, beggary, increasing unemployment, crime, cultural incompatibility (Asadi-Lari et al., 2010).

Development of urbanization and industrial activities in major cities lead to wide changes in profile of important parameters, such as health, economy, and social phenomena. Also, another significant effect of urbanization is urban heat island (UHI) caused by climate change, air pollution, and release heat in the atmosphere (Zanjani, Shadpoor, Mirzaie, & Mehriar, 2004).

Apartment-living, as a progressive subset of modern urban lifestyle, is one of the attractive subjects to many of Iranian families in recent years. Due to population growth and necessity for housing, the simple and intimate non apartment house has been replaced with modern small apartment unit which causes different outcomes and challenges in different aspects of health (Aryanpour, 2006). Nowadays, apartment-living, developing in high speed is considered as an integral part of urban lifestyle, while this manifestation of new urbanism appears to be the reason of social isolation, various crimes, reduction in privacy and less opportunity for children play in a safe environment (Burridge & Ormandy, 2013).

Some epidemiological studies have revealed a direct relationship between reduction of mental health status and height of multi-room apartment from the ground level (Evans, Wells, & Moch, 2003). According to endocrinologists, men and women living in an apartment in major cities have suffered from the vitamin D deficiency due to the following reasons: **i)** Loss of backyardcauses loss of exposure to direct sunlight; **ii)** Reduction to use food containing vitamin D. Also, according to different experts’ opinions, apartment-living and weight gain are closely associated to each other (Evans et al., 2003; Sanders, Bowie, & Bowie, 2004).

As Sara Sanders et al. have showed that more than 70% of people living in an apartment in Miami were suffered from lack of mental health, social and physical well-being; therefore, they decided to return to their previous non apartment houses because of intense pressure originating from living in an apartment building (Sanders et al., 2004). A study by Kamran et al. have revealed that despite of many social contacts in major cities, relationship seems to be formal and empty of compassion. Their finding has showed that there is a significant difference in social compatibility between apartment-living and non-apartment-living residents (Kamran, Hosseini, & Zabihinia, 2009).

Of course, phenomenon of apartment-living by itself cannot be considered as health risk factor, yet interaction with other factors escalate negative effects on different aspects of health of residents. According to this explanation, it is necessity to evaluate the reasons for creating and increasing the phenomenon, known as the symbol of modern life, in order to enforce best management in development approach. These potential effective variables are adapted of socio-economic and demographic factors. However, after extensive research, we did not find any study regarding the predictive role of socio-economic and demographic factors for apartment-living or in broader level, urbanization. Considering progressive development of apartment-living and potential effects of this modern lifestyle remaining on health of residents; in addition, according to the motto of the World Health Organization (WHO) on the World Health Day in 2010, “urbanization and health” this research aimed to apply the simplest statistical techniques in order to predict the rate of apartment-living based on socio-economic and demographic factors, and to present overall summary in the field of sociological interaction of these variables by approach to different aspects of health.

## 2. Material & Methods

### 2.1 Study Design and Participants

This descriptive-analytical study was performed on 600 apartment and 800 non-apartment residences using multi-stage cluster sampling techniquein the city of Shiraz, Southern Region of Iran, during the year 2011. The inclusion criteria were for all participants as follows: i) living in a complex with more than 10 apartments at least for two years ii) aged more than 15 years.

### 2.2 Sampling Process

The sample size is based on comparing mean of two independent groups sample size formula performing in an initial pilot-study on 40 apartment and non-apartment residents, considering α = 5% and β = 20%, and Standard deviation for health score of any aspect is approximately to be 2.5 and at least 0.5 unit difference for health score of any aspect, and also, in order to adjust the effect of process of multi-stage cluster sampling, with design effect equal to 2, because of potential of lack of cooperation as well as increase power of study for any kind of house (apartment or non-apartment) are calculated 400 households.

In the main sampling procedure in this study was initially decided not to include the information and the data obtained through pilot-study. The locations included in pilot-study were also considered in the main sampling procedure; however, the apartment complexes included in the pilot–study were not considered in the main sampling procedure.

To apply the sampling procedures in main study similar to pilot-study, among all regional divisions (clusters) in the city of Shiraz performed by the Shiraz municipality, five divisions were chosen using a random sampling and a table of random number. It is noted that the city of shiraz selected for pilot-study by easy and convenience sampling method due to near and familiar location as well as easy commute for the researcher.

In the next step, for the total volume of sample size which is normally 400 households for any kind of residential, we assessed 80 households for any kind of residential. So, in every selected division, four apartment complexes were chosen through convenience method, followed by selection of 20 households through random sampling and a table of random number. Also, in order to comparison, 20 households of non-apartment resident were chosen through convenience method in the same area of selected complexes. In every household, all people above 15 years of age were reviewed, and then a questionnaire was completed.

The inclusion criteria were only assigned for people above 15 years of age due to lack of proper questions for physical, emotional, social and environmental features of children and adolescence below 15 years of age.

Considering the volume of samples and applying cluster sampling method, in the beginning, sampling procedure was performed with considering the design effect=2, in order to adjust the variance resulted from sampling as well as its effect on our findings. Since our sampling size was large enough, for accurate measurement of sampling effect on obtained result, the linearization method and hierarchical cluster analysis were applied using Stata 10 software to illustrated that there was no skewnessin distribution of health score among the variables (p=0.0.1).

### 2.3 Instrument

In this study, based on the specific objective for determining the different aspects of health of urbanization lifestyle with approach to different aspects of health, the standard questionnaire of World Health Organization Quality of Life-BREF (WHOQOL- BREF) was applied. This questionnaire is a tool to collect information about quality of life, generally. Psychometric features of this questionnaire, assigned for Iranian population, revealed that the form could be as a proper tool in our study due to the validity, reliability and ability of appropriate response to our questions (WHOQOL-BREF Group, 1993; World Health Organization, 1996; WHOQOL-BREF Group, 1996).

WHOQOL-BREF contains 24 questions regarding the following subjects: physical health (7 questions), psychological health (6 questions), social relationship (3 questions) and environmental health (8 questions). Also, there are two extra questions to assess health condition and quality of life, in general, and then the points were calculated, separately. So, there is total of 26 questions in this form (Nejat, Montazeri, Holakouei Naeeni, & Mohammad, 2006).

### 2.4 Variables

The participants had to use their experiences in recent 4 weeks to answer the first 24-question of the form. In this study, the following variables were evaluated as predictors: family size, age, gender, education level, marital status, occupation, monthly income, type of house ownership, home area per person (70-90 square meters Person), duration of residency, economical level of location, having chronic or acute disease (healthy-chronic, acute or chronic-acute), and living lonely as an indicator for social-economic and demographic factors. Living lonely; however, is considered as a crude indicator of social-well-being. Also, some studies have introduced home area per person as an alternative economic indicator (Nejat et al., 2006).

Different study sub-groups were formed based on the collected data from the above-mentioned variables. From age perspective, six sub-groups were assigned as follows: **i)** 15-25 years old, **ii)** 26-35 years old, **iii)** 36-45years old, **iv)** 46-55 years old, **v)** 56-65 years old, and **vi)** > 66 years old. From gender and occupation perspectives, two sub-groups were assigned as follows: woman and man sub-groups, as well as employed and unemployed sub-groups, respectively. From educational level perspective, they were divided into four sub-groups as follows: **i)** illiteracy, **ii)** first-eighth grade, **iii)** ninth-twelfth grade, and **iv)** >diploma level. Four sub-groups were also considered based on marital status as follows: **i)** single, **ii)** married, **iii)** divorced, and **iv)** widowed. In the term of family size, three sub-groups were formed as follows: **i)** two-person, **ii)** three- or five-person, and **iii)** >five-person. From health status perspective, there were four sub-groups as follows: **i)** healthy, **ii)** chronic illness, **iii)** acute illness, and **iv)** chronic-acute illness. There were three sub-groups from monthly income perspective as follows: **i)** high monthly income, **ii)** average monthly income, and **iii)** lowmonthly income. Finally, for house ownership perspective, participants were divided into owner and renter sub-groups.

### 2.5 Analysis Method

After collecting data from mentioned-variables of two main study groups, they were entered into the logistic regression equation (Formula (1)), followed by applying SPSS16 for variance analysis. Also, after excluding missing data, forward stepwise method (conditional) was carried out to analyze the data. The mean of all collected scores of different aspects of heath in differentiation of apartment-living was calculated as crude value, and then the obtained results can be compared two by two using two-sample t-test. It was followed by presenting the adjusted scores using linear regression analysis in order to assess the findings of variables. In all tests, Significance level was p<0.05.


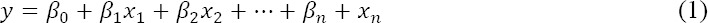


### 2.6 Ethical Consideration

Ethical approval was obtained from Kerman University of Medical Sciences Research Committee. So, the participants were given written and oral information about the study. They were anonymous. All of them participated willingly and voluntarily in this study. All the participants were informed of the study objectives and how to perform it. In the other hand, In regard to implementation of Ethical consideration, with respect to grouping data analysis and necessity of access to individual confidential information, in initial referral to families, the oral satisfaction of cases was to catch by researcher and however, if any family didn’t interested for participate in the project, it isn’t any compulsion and we selected required cases at close neighbor families.

## 3. Results

In this study, the apartment-living group included 600 participants (386 men and 214 women) showed overall mean ±SD, mod, and median of age sub-group to be 31.38±1.62, 29 and 27 years old, respectively. In addition, non-apartment-living group included 800 participants (590 men and 210 women) revealed mean ±SD, mod, and median of age sub-group to be 32.83±1.42, 30 and 29 years old, respectively. For age sub-group, no significant difference was detected between means of apartment-living and non-apartment-living groups (p<0.05).

The range age of the participant was 15-76 years old. Table 1 shows that descriptive characteristics of two study groups based on the mentioned-variables. Probability of multi-co linearity among the predictor variables was assessed before entering the dataof social-economic or demographic factors into regression equation. Since none of correlation coefficients were higher than 0.7015, there was no probability of multi- co linearity among the obtained data.

**Table 1:** Frequency of cases basis upon levels of variables in groups

Variables		Un Apartment- living frequency (%)	Apartment- living frequency (%)
Gender*	Male	386(64.3)	590(73.8)
	Female	214(35.7)	210(26.2)
Job*	Employed	515(85.8)	595(74.4)
	Un-employed	85(14.2)	205(25.6)
Literacy (year)	Unread*	32(5.3)	89(11.1)
	1-8*	156(26)	256(32)
	9-12	282(47)	385(48.1)
	>12*	130(21.7)	70(8.8)
Marital status	single	39(6.5)	44(5.5)
	Married*	362(60.3)	575(71.9)
	Divorced widow*	118(19.7)	125(15.6)
	Died widow*	81(13.5)	56(7)
Disease	Healthy*	340(56.7)	511(63.9)
	Chronic	83(13.8)	100(12.5)
	Acute*	136(22.7)	126(15.8)
	Chronic-acute	41(6.8)	63(7.9)
Family size	2-person*	326(54.3)	137(17.1)
	3-5 person*	211(35.2)	488(61)
	>5 person*	63(10.5)	175(21.9)
Age (year)	15-25	84 (14)	93 (11.6)
	26-35	145 (24.2)	209 (26.1)
	36-45*	134 (22.3)	259 (32.4)
	46-55	107 (17.8)	119 (14.9)
	56-65*	86 (14.3)	73 (9.1)
	>66	34 (7.3)	47 (5.9)
house ownership (family unit)*	Owner	108 (27)	276 (69)
	Renter	292 (73)	124 (31)
	Low*	127 (31.7)	50 (12.5)
Monthly income	Average	205 (51.2)	270 (67.5)
	High	68 (17)	80 (20)
living lonely*	Alone	22 (3.6)	11 (1.3)
	With family	578 (96.3)	789 (98.6)
23.62-106.25 (mean=64.9)	12.5-37.5 (mean=25)		Home area perperson

* Differences is significant (p<0.05).

In order to determine the effect of different variables mentioned above as predictors on apartment-living as criterion variable, all variables entered in multi-variable logistic regression model. All obtained results of multi-variable logistic regression are demonstrated in Table 2. Also, chi-square test confirmed obtained results of multi-variable logistic regression were significantly different (p=0.001).

**Table 2:** Results of logistic regression model between apartment- living with socio- economic and demographic variables in final step

STEP		B	S.E.	df	Sig.*	Exp(B)	CI95% FOR Exp(B)
Age	15-30	-	-	1	0.001	-	-
	>30	-0.38	0.003	1	0.001	8.31	8.43- 7.92
Gender	Male	-	-	1	0.001	-	-
	Female	18.7	0.23	1	0.001	0.64	0.69- 0.51
Job	Un employed	-	-	1	0.001	-	-
	Employed	45.68	0.39	1	0.001	6.9	7.3- 6.1
Literacy	Unread	-	-	4	0.001	-	-
	1-8	48.97	0.001	1	0.001	1.86	2.11- 1.23
	9-12	31.52	0.002	1	0.001	4.9	5.21- 4.1
	>12	-23.16	0.26	1	0.001	1.14	1.94- 1.02
Monthly income	<3000000 Rials	-	-	2	0.001	-	-
	3000000-6000000 Rials	0.36	0.001	1	0.001	6.57	6.86- 6.12
	> 6000000 Rials	0.49	0.05	1	0.001	9.49	9.69- 9.11
Marital status	single	-	-	3	0.001	-	-
	Married	-27.48	0.02	1	0.001	8.64	8.71- 8.22
	Divorced widow	48.08	0.03	1	0.001	7.64	7.78- 7.1
	Died widow	8.51	0.06	1	0.001	42.66	42.83- 41.98
disease	Healthy	-	-	3	0.001	-	-
	Chronic	-23.08	0.46	1	0.001	0.0001	-
	Acute	-23.29	0.48	1	0.001	0.0001	-
	Chronic-acute	-22.30	0.51	1	0.001	0.0001	-
Family size	2-person	-	-	2	0.001	-	-
	3-5 person	-5.3	0.36	1	0.001	0.005	-
	>5 person	-1.8	0.03	1	0.001	0.16	0.29- 0.12
house ownership	Owner	-	-	1	0.001	-	-
	Renter	3.45	0.41	1	0.001	8.73	8.89- 8.23
living lonely	With family	-	-	1	0.001	-	-
	Alone	0.86	0.04	1	0.001	1.39	1.58- 1.20
Home area per person	-	-0.99	0.48	1	0.001	0.98	1.1- 0.9
Constant	-	-115.38	0.16	1	0.001	0.102	0.21- 0.09

* Differences is significant (p<0.05).

In this model, crude and adjusted values of different variables were compared. For age variable, rate ratios (OR) and adjusted value are 1.63 and 8.31, respectively. The high amount of adjusted value represents existence of confounding effect of other variables in multi-variable model. This shows the requested rate of apartment-living in age sub-group above 30 years in comparison to age sub-group of 15-30 years is 8.31 times more.

For gender variable, OR and adjusted value are 0.0001 and 0.64, respectively. The difference in adjusted value represents existence of confounding effect of other variables in multi-variable model. This reveals rate of apartment-living in women sub-group in comparison to men sub-group is 0.64 times more.

OR and adjusted value for occupation variable are 2.08 and 6.9, respectively, while this difference also represents confounding effect of other variables. This reveals rate of apartment-living in employed sub-group in comparison to unemployed sub-group is 6.9 times more.

OR for marital status perspective is calculated 0.73; however, adjusted value of this variable reveals rate of apartment-living in married, divorced and widowed sub-groups in comparison to single sub-group are 8.64, 7.64 and 42.66 times more, respectively. The high amount of adjusted value in widowed sub-group confirms existence of a strong confounding effect among independent variables.

OR of educational level perspective is calculated 0.66. Meanwhile, adjusted value of this variable reveals rate of apartment-living in first-eighth grade, ninth-twelfth grade, and overdiploma level sub-groups in comparison to illiterate sub-group are 1.86, 4.9 and 1.14 times more, respectively.

For chronic or acute disease perspective, OR value is 0.88, while adjusted value of this variable reveals rate of apartment-living in chronic, acute, and chronic-acute sub-groups in comparison to healthy sub-group is 0.0001 times more in all three groups. The differences of these effects in single or multi-variable model also represent existence of confounder among variables and their confounding effects.

OR value for family size perspective is 3.12, while adjusted value of this variable reveals rate of apartment-living in three- or five-person andoverfive-person sub-groups in comparison to two-person sub-group is 0.005 and 0.16 times more, respectively. Variables, like duration of stay and place of residence are not significantly different due to economic status of residence in this study, which it confirms they are not considered as proper predicated variables for apartment-living group.

For monthly income perspective, OR value is 5.36, while adjusted value of this variable reveals rate of apartment-living in average monthly income and high monthly income sub-groups in comparison to low monthly income sub-group are 6.57 and 9.49 times more, respectively.

For house ownership perspective, OR and adjusted values of this variable reveals rate of apartment-living in owner sub-group in comparison to renter sub-group are 5.093 and 8.73 times more, respectively.

For home area per person perspective, the result shows that per one square meter increase in home area, rate of apartment-living increases 0.98 times more.

Finally, for living lonely perspective, the obtain result of this variable shows that OR and adjusted value for people living alone in comparison to people living with their families are 2.33 and 1.38 times more, respectively.

Value for coefficient of determination (*Cox & Snell* R^2^) of this model is calculated 0.8199, being as an appropriate odel. Almost 82% of changes in response variable to rate of apartment-living are justified bypredictor variables or socio- economic and demographic factors. Observation of the likelihood of this sectionwith respect to reduction of the scores-*2Log Likelihood*- illustrates that the model in every forward-stepcompared to previous-step is improved.

Variables, like age, education level, marital status, health status, family size and home area reveal inverse relationship with rate of apartment-living; however, remaining variables show direct relationship with rat apartment-living.

In addition, investigation on different aspects of health shows the results based on crude values for apartment-living and non-apartment-living groups, respectively, as follows: mean score of physical health are 13.57±3.47 and 16.41±2.99, mean score of mental health are 10.71±2.97 and 14.87±2.72, mean score of social health are 8.57±3.53 and 13.84±2.98, and mean score of environmental health are 13.59±3.73 and 10.18±3.52 (p<0.0001). After adjusting the linear regression model for gender; educational level; marital status, age; occupation; family size; monthly income; health status; ownership; and home area, the values for apartment-living and non-apartment-living groups, respectively, are reported as follows: in physical aspect are 14.41 and 15.61, in mental aspect are 12.6 and 14.47, in social aspect are 8.74 and 13.72, and in environmental aspect are 15.42 and 9.23. So, health scores of different aspects for both study groups show significant differences (p<0.0001); we also observed a significant difference in health score between two sub-groups among fferent aspects (Graph 1).

**Figure 1:**
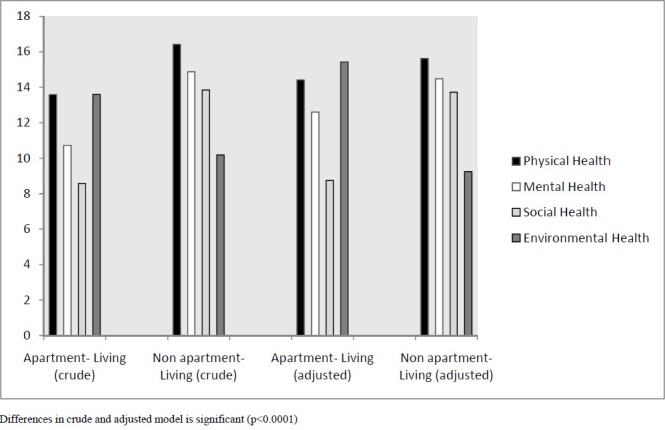
Changing score of health dimensions basis upon apartment- living

Finally, the following analysis is presented based on the different variables of social-economic and demographic factors to predict the request or probability rate of apartment-living:

Apartment living (dependent variable) = - 115.38 – 0.38 (age) + 18.7 (sex) + 45.68 (job) + 48.97 (1-8 years literacy) + 31.52 (9-12 years literacy) – 23.16 (> 12 years literacy) + 0.36 (3000000-6000000 Rials income monthly) + 0.49 (> 6000000 Rials income monthly) – 27.48 (married) + 48.08 (divorced widow) + 8.51 (spouse died widow) – 23.08 (acute disease) – 23.29 (chronic disease) – 22.30 (acute- chronic disease) – 5.3 (3-5 person family) – 1.8 (> 5 person family) + 3.45 (home ownership) + 0.86 (live single) – 0.99 (home area).

ANOVA test results show significant differences among the different scores in scales of social-economic and demographic risk factors which it was based on risk factors happening for apartment-living group. Therefore, following results in Table 3 obtained from different sub-groups of apartment-living group are associated with high level of risk factors.

**Table 3:** ANOVA test results among the different scores in scales of social-economic and demographic risk factors apartment-living

Sub group	F	df	P-value*
Men	7.92	(1,1394)	p<0.001
Over 30 years	38.42	(1,1394)	p<0.001
Employed	34.25	(1,1394)	p<0.001
1-8 years literacy	42.11	(1,1394)	p<0.01
9- 12 years literacy	22.98	(1,1394)	p<0.001
>12 years literacy	75.23	(1,1394)	p<0.01
average monthly income	45.36	(1,1394)	p<0.001
high monthly income	50.34	(1,1394)	p<0.001
Married	30.92	(1,1394)	p<0.001
Divorced widow	76.12	(1,1394)	p<0.01
Died Widow	48.23	(1,1394)	p<0.01
Acute disease	6.75	(1,1394)	p<0.01
Chronic	9.37	(1,1394)	p<0.001
chronic- acute	11.37	(1,1394)	p<0.001
family size of 3-5 persons	52.74	(1,1394)	p<0.001
family size of >5 persons	112.22	(1,1394)	p<0.001
house ownership	93.29	(1,1394)	p<0.001
living lonely	117.39	(1,1394)	p<0.001

Differences is significant (p<0.05).

## 4. Discussion

The result of this study shows that volume effects of variables, including age; occupation; marital status; educational level; monthly income; and home ownership in scales of social-economic and demographic factorsregarding the rate of apartment-living are relatively high. Considering high rate of apartment-living, economic and demographic factors remain stronger effects than social factor. Also, average of health scores in physical, mental, social and environmental aspects in two study groups reveals statistically significant differences. The overall average scores of different aspects of health, except environmental aspect in apartment-living group, are significantly less than non-apartment-living, even after adjusting for confounding variables.

The result of our study also demonstrated that overall health status in apartment-living was lower than those in non-apartment-living. In analyzing of different aspects of health among variables, like educational level; health status; monthly income; and age because of common adjusted health score among them and volume of effect remaining form them, they are not considered as an important confounding factor for fluctuation of health aspects in apartment-living group. In other words, mean of health score in apartment-living group in all aspects after adjusting is increased, except environmental aspect which is significantly lower than non- apartment-living group.

In addition, adjusting the scores by confounding variables resulted to differences among physical, mental, social, and environmental aspects between original and adjusted models as follows: 0.84, 0.48, 0.17 and 1.83. For apartment-living group, fluctuation of health aspects for physical, mental, social and environmental aspects in original model, without considering confounding variables, were reported respectively as follows: 2.84, 3.29, 5.27 and 3.41. Removing the confounding variables causes differences between original and adjusted models as follows: 0.84, 0.48, 0.17 and 1.83. This confirms that net share resulted of confounding variables in difference of scores among health aspects are as follows:0.84, 0.48, 0.17 and 1.83, while rate of apartment-living in physical, mental, social and environmental aspects are as follows: 2, 2.81, 5.1 and 5.24.

The most effective variables is observed in environmental aspect, and then in physical, mental and social aspects. After adjusting, the gaps of scores among all aspects of two main groups, except environmental aspect, were decreased, so the scores were closertogether. Between two studied groups, the closest relationship is between physical aspects and least relationships are between social aspects and mental aspects, respectively.

Obtained result in environmental aspect is different because adjustment causes to raise the crude value in apartment-living group, as well as to increase differencesbetween environmental aspects of two groups. Therefore, health scores of three aspects (physical, mental and social) in apartment-living group increase due to elimination of confounding variables, which it confirms the difference between two groups is less.

The obtained result also reveals that health score of environmental aspect increases, confirming different between two groups is more. Consequently, after adjusting and elimination of confounding variables, a difference between two groups in physical, mental and social aspects are less, but in environmental aspect is more. This research also found that variables, like: age; health status; family size; educational level over twelfth grade; married; and house area are inversely associated to rate of apartment-living, while the rest of variables, including gender; occupation; education level of first-eighth grade and ninth-twelfth grade; home ownership; monthly income; and living lonely are directly associated to rate of apartment-living. In the other hand, Thomson et al. (H. Thomson, Petticrew, & Douglas, 2003), Vivian et al. (Loftness, Hakkinen, Adan, & Nevalainen, 2007), Baker et al. (Baker, 2010), Vibha et al. (Vibha, Saddichha, & Akhtar, 2010) and Thomson G et al. (G. Thomson et al., 2005) each obtained significant association in their studies between apartment-living and health status that is similar with our approach to healthy effect of design of living- place (apartment or non-apartment). Therefore, we can imagine a direct association between apartment-living and Socio-Economic and Demographic Factors with healthy status of residents.

The reasons behind the inverse association of some of variables can be easily justified. For example, for age variable, we assume that apartment-living is dominant lifestyle of young couple in comparison with elderly couples of society. Most of young couples attempt to have family at young age and housing is their first agenda, so they have higher tendency to live in an apartment. However, most elderly couples prefer to live near their children. It is also noted that there is higher rate of apartment-living in older age because young people are usually single and have less tendency for housing in comparison to married couple.

About inverse association of educational level over twelfth grade with rate of apartment-living, it seems that higher education people are more likely to live in an apartment; however, they, nowadays, suffer from low purchasing power and high rental expenses. Also their awareness is increased about health side effects of modern lifestyle. So, these strong reasons decrease their tendency for apartment-living. Tendency of people with educational level below twelfth grade to live in an apartment is loss because of not having academic education, having improper occupation, having poor economic status, and low power of purchasing or renting an apartment unit. Therefore, from the low level of education prior to university, tendency for apartment-living rises, but right before university, this path reverses and follows a descending path.

A study by Tsai et al. about home-preferred choice among psychiatric patients have revealed that single and lonely people are more likely to live in an apartment because this request greatly depends on their mental health conditions, overwhelmed by living in an apartment (Tsai, Bond, & Davis, 2010). This study is accordance with our study confirming higher rate ofapartment-living among single and divorced sub-groups in comparison to married sub-group. In single lifestyle, there is higher probability of illegal drug usage or involve in illegitimate relationship. In addition, different studies have shown poor mental health condition of people living in an apartment which it leads them to different social misbehavior (Amann, 1991; Cheng, T. H. Chen, C. C. Chen, & Jenkins, 2000; Johnsson Fridel et al., 1996; Hirschfeld et al., 2000; Treena, Wu, & Angelique Cha, 2011).

It is noted that we also observed direct association between living lonely sub-group and rate of apartment-living. From health status perspective, people with acute or chronic illnesses face undesirable economic condition, so they expose environmental hazards causing severity of illness more than people with suitable economic condition.

In public, apartment-living style is limited to wealthy and privileged class with specific occupation; therefore, based on this belief, apartment-living is symbol of healthy lifestyle. Regarding the family size perspective, whenever family size grows, the rate of apartment-living decreases, while two-person family size has more tendency to live in an apartment. The explanation of family size perspective is the same as the age perspective. Briefly, we may conclude that apartment provides a suitable lifestyle in the life today for young couples preferring fewer numbers of children and facing financial difficulty. However, next to an apartment, there is always a non-apartment house having more space per person with more household members which displays the inverse relationship between home area and apartment-living.

According to these interpretations, we could illustrate the direct association of some variables, such as male, occupation, monthly incomes, and living lonely with rate of apartment-living. Utilizing extensive research from various sources, applying accurate and appropriate keywords, and considering wide variables assisted us to present the first descriptive cross-sectional study in this subject to discuss the prediction of rate of apartment-living based on social-economic and demographic factors.

Lack of other searches and studies in this field in order to compare with our findings are considered to be the limitations in this study. Therefore, as baseline study, we only tried to explain the possibility of finding in a reasonable limit.

Based on the obtained result, it is very important that policy makers in urban areas consider the determinative role of socio-economic and demographic factors, which are involved in selecting apartment-living lifestyle by urban residents. We also recommend considering the influence of education in reduction or modification of lifestyle or pattern of residents in apartment-living areas in future studies; in addition, other evaluations of different aspects of apartment-living lifestyle may be as a complementary source beside our study.

## 5. Conclusions

Wherever the mean of different dimension of health scores are lesser in apartment living residents even after adjust confounder variables, it could be said that unprogrammed apartment living could be an potential challenge for health of urban residents (Cohen, 2004). So, it is very important to consider spreader factors of this challenge that the demographic and social- economical variables are there from. Clarify of these variables and their effect size on probability rate of apartment living could help to politicians for programming to decrease health effects of apartment living that is inevitable subject in current world modern life. Also, it is considered that unplanned urbanizationhas caused negative effect on health of individual and society. However, with modification and optimization of different socio-economic components, a healthy stable society is achievable. So, it is about the time of pay attention to the primary prevention of initial impact of apartment-living, and future health policy must be facilitated, specifically for urbanization and apartment-living.
